# Patient and Provider Perspectives on a Decision Aid for Familial Hypercholesterolemia

**DOI:** 10.3390/jpm8040035

**Published:** 2018-11-04

**Authors:** Medhat Farwati, Ashok Kumbamu, David C. Kochan, Iftikhar J. Kullo

**Affiliations:** 1 Department of Cardiovascular Medicine, Mayo Clinic, Rochester, MN 55905, USA; farwati.medhat@mayo.edu (M.F.); kochan.david@mayo.edu (D.C.K.); 2 Center for Science of Health Care Delivery, Mayo Clinic, Rochester, MN 55905, USA; kumbamu.ashok@mayo.edu

**Keywords:** patient decision aid, shared decision-making, familial hypercholesterolemia, focus group discussions

## Abstract

Familial Hypercholesterolemia (FH) is an inherited disorder associated with increased cardiovascular risk that requires patients to make multiple impactful decisions regarding the management of their condition. Patient decision aids (PDAs) can facilitate shared decision-making (SDM) and enable patients to make choices that are concordant with their goals and values. To inform the development of a PDA for patients with FH, we employed a qualitative inductive approach and focus group discussions with patients, physicians, and genetic counselors. We explored and analyzed the perceptions and understanding of these stakeholders related to a PDA for FH and identified important concepts to include in the PDA. Categories emerging from focus group discussions included: (a) perceptions of a PDA related to FH; (b) discussion about the content of a PDA related to FH; and (c) SDM. In general, stakeholders were in favor of developing tools which can inform and individualize discussion about genetic testing and treatment options for FH. Physicians valued a tool that facilitates knowledge transfer to FH patients. Patients desired a tool to help them understand the genetic aspects of and treatment options related to FH. Genetic counselors emphasized the inclusion of visual aids to support discussion with patients. Potential barriers to and facilitators of PDA implementation were identified. The input of various stakeholders will inform the development of a prototype tool that will be iteratively tested before implementation in the clinical setting.

## 1. Introduction

Familial hypercholesterolemia (FH) is a relatively common inherited disorder of lipid metabolism with a prevalence of 1 in 250 [1]. It is estimated that in the United States, 0.65 to 1 million patients have FH but of these less than 10% carry the diagnosis [2,3]. Thus, FH is underdiagnosed and consequently undertreated [4]. Elevated low-density lipoprotein cholesterol (LDL-C) levels can increase the risk of premature atherosclerotic cardiovascular disease (ASCVD). In untreated heterozygous FH, premature death or myocardial infarction can occur at a young age [5]. However, with appropriate treatment life expectancy can be extended to normal [5].

An internet-based survey conducted by the American College of Cardiology among patients enrolled in Mended Hearts—one of the largest peer-to-peer heart patient support networks in the US—showed that 57% of patients were not at all aware of FH and only 5% reported that they were well aware of the disease [6]. This is even though patients are generally aware of having high cholesterol or a family history of high cholesterol. Given the lack of knowledge about FH among patients [7], there is a need for patient-centric tools to help patients make decisions related to drug therapy, genetic testing, and screening of family members [8].

Shared decision-making (SDM)—a frequently advocated approach for patient-centered care [9,10,11]—can be defined as a collaborative process for arriving at healthcare decisions informed by the best available evidence provided by clinicians as well as the patient’s values, goals, and preferences [12]. Various approaches have been proposed to advance implementation of SDM in the clinical setting [10].

Patient decision aids (PDAs) are being increasingly used to foster SDM and enable patients to make choices that are concordant with their goals and values. PDAs provide the basis for SDM by educating the patient about the condition, especially about treatment and screening options with the risks and benefits of those options presented in an unbiased way [13,14,15]. A Cochrane review of 200 studies using decision aids found that compared to regular care, decision aids result in greater patient involvement, more realistic expectations, and a greater level of knowledge regarding clinical decisions [15]. The American Heart Association’s scientific statement on FH highlights the need for decision aids for patients with FH [8]. To our knowledge, however, such tools have not yet been developed.

While several theoretical frameworks can be used to create PDAs, most developers regard the involvement of relevant stakeholders early on in the development process as a crucial initial step [16]. Employing a qualitative inductive approach and focus group discussions with patients, physicians, and genetic counselors, we explored and analyzed perceptions and understandings of these stakeholders about a PDA pertaining to FH. We also aimed at identifying themes or topics that clinicians and patients want to incorporate in a PDA for FH.

## 2. Materials and Methods

This study was approved by the Mayo Clinic Institutional Review Board and took place between April 2017 and November 2017. Informed consent was provided by all participants of this study.

### 2.1. Participants and Recruitment

Five focus groups were conducted: two with physicians, two with patients, and one with genetic counselors. Each focus group lasted approximately 60 min and at least four participants were present in each group discussion. We used a convenience sampling and criterion-based approach [17] to invite consultants in the Mayo Clinic Network who see patients with FH in an outpatient setting. Physicians and genetic counselors at the Mayo Clinic-Rochester campus were invited by email.

Patients were recruited by postal mail. The inclusion criteria for patients were: (1) adults >18 years of age meeting the Dutch Lipid Clinic Network (DLCN) criteria for FH; (2) living within 50 miles of Rochester, Minnesota; and (3) without major barriers to providing informed consent (i.e., dementia, severe hearing, or visual impairment).

### 2.2. Data Collection

To facilitate focus group discussions, a semi-structured moderator guide was developed based on the existing literature related to perceptions of PDA. In the moderator guide, we crafted open-ended questions to allow the participants to share their opinions and perspectives on FH without any influence of researchers. Topics explored in focus groups were: counseling patients with FH, patients’ experiences with FH, and recommendations for creating a PDA for FH. Sample questions are summarized in Table 1 and Table 2. Two qualitative researchers (AK and MF) co-moderated the discussions and notes were taken in all sessions. All focus groups were audio recorded, transcribed verbatim, and anonymized to protect the identity of participants. Lunch and compensation of $25 in the form of a gift card was provided to participating patients. Clinicians received a $100 honorarium. This is consistent with previous studies involving focus group discussions with clinicians. This amount was not considered significant enough to cause bias in the clinician’s opinions.

### 2.3. Data Analysis

Data collection and analysis were guided by standard qualitative inductive approaches [18]. Two qualitative researchers (AK and MF) coded transcribed data. In the initial phase, a codebook was developed based on open reading of two transcripts, the moderator guide, and research aims. Once the codebook was finalized, it was used to code all the transcripts independently. While focusing on a priori codes, codes emerging from the data that were not identified in advance were also considered. Any disagreements over assigning and labeling of codes were resolved by discussion at a biweekly research meeting.

The coding process used principles of grounded theory methodology such as open, axial, and selective coding approach. Nvivo software (QSR International, 2008) for data management and analysis was used [17,19]. Open coding involved reading the transcripts and labeling the ideas that emerged during the discussion. Axial coding ensued, namely placing these labels into broad themes based on their relationship. In the selective coding phase, themes were reorganized to develop a tool that could be further refined, evaluated, and used in a clinical context.

## 3. Results

Of the 365 physicians (213 cardiologists, 94 internists, and 58 family medicine physicians) who met the inclusion criteria, 12 agreed to participate in the study. Of the six genetic counselors who were invited, five participated in the discussion. Demographic characteristics of the clinicians interviewed are summarized in Table 3. Of the 134 patients invited, 28 agreed to participate in the study and 14 actually attended the discussions. Demographic characteristics of the patients interviewed are summarized in Table 4.

Based on analysis of transcripts, the following key categories were identified: (a) perceptions of a PDA related to FH; (b) discussion about the content of a PDA related to FH; and (c) shared decision-making.

### 3.1. Perceptions of a PDA Related to FH

Participants discussed their perceptions of a PDA for FH. Two themes emerged from the discussions: facilitators of and barriers to integration of PDAs in the clinical workflow.

#### 3.1.1. Facilitators of Implementation

Participants highlighted potential factors that could facilitate the integration of PDAs into clinical encounters with FH patients. Many participants emphasized that for a PDA to be most useful, information should be presented in a layperson format. From a patients’ standpoint, it is of paramount importance to have an introductory section that provides a brief description of FH and explains the genetic components of the disease to the patients in a simplified, yet succinct manner. One patient stated:

“*I know it would be probably good to have a very simple DNA, […] like show a lineage of how in a family it might go to make it more visible to someone that hasn’t, isn’t familiar with it at all. And yeah probably a real simple diagram or something that shows the links*” (Patient FG I).

Resonating with the patients’ viewpoint, a common notion that emerged from physicians’ discussion was that a concise tool without technical medical jargon would enhance communication with patients. Reflecting this, one physician explained:

“*Just make sure that things like that are again worded in such a way that we are doing the most effective communication with our patients as opposed to (us) just explaining medical jargon to them so they can speak our language*” (Physician FG I).

Physicians also noted that the usefulness of decision aids can be maximized by making them accessible to patients not only during time-limited clinical encounters but also in their homes. One physician stated:

“*Especially if they can take it home, I think that would be very effective because they can kind of re-look at that information then. I think just having this on my computer so I can navigate it isn’t going to be enough*” (Physician FG I).

Genetic counselors echoed similar thoughts, noting that before deciding, patients would need time to process all the information presented during the consultation. Patients would benefit to a greater extent if they have access to the tool both before and after counseling sessions. One genetic counselor stated:

“*We’re hitting them [patients] with a lot of information that they were not necessarily prepared to digest. So I think that’s where again having that tool available maybe pre-consult but also post-consult is something that would be helpful for them to prepare for their visit. But, also if they aren’t making a decision to be able to go back and help them with decision-making afterward*” (Genetic Counselor FG).

Another genetic counselor also emphasized this concept:

“*I think too the tool would be great if the patients could access it after the session because if they don’t make a decision right then […] they’ll go home and think about it, it’s hard to remember really what you talked about*” (Genetic Counselor FG).

Many participants also emphasized the importance of the design of the tool that enables successful implementation. A genetic counselor highlighting the importance of the integration of relevant visual aids into the tool said:

“*I think visual aids are always something that can be helpful for patients to really see where they’re at*” (Genetic Counselor FG).

#### 3.1.2. Barriers to Implementation

Participants discussed many aspects related to the implementation of the tool in the clinical setting and identified several potential barriers that may hinder effective uptake of the tool. Participants from each group expressed their opinions and concerns about potential barriers from their understanding of and experience with other PDA tools.

For physicians, time is a paramount factor that could potentially be a major barrier. They were concerned about working under time pressure in the clinic. One physician noted:

“*I don’t see any time in our clinical time where we have the extra 15 min that this- if we opened this bag of worms saying Oh, there’s a genetic test. That means okay now you better sit down for another 20 min and discuss with me what this means. I don’t have that leeway in*” (Physician FG II).

Another factor that physicians were concerned about was their limited clinical experience as well as expertise in managing FH patients. Reflecting on these situations a physician openly stated:

“*If you’re going to be doing shared decision-making, you need to be super comfortable with the material that you’re having. So, if this is something that you’re going to be doing once a month, probably by the next time you don’t even remember where the link is or where are the papers. On the other hand, if you do it all the time like I can have I can do that for the things I see in my clinic with the eyes closed. But, I will probably be more comfortable sending the patients to giving them something and say, “Here, you can read this, but I’m referring you to the Lipid Clinic or to the genetic counseling because I feel I will be struggling*” (Physician FG II).

From a physicians’ pragmatic perspective, another potential barrier is having to deal with multiple comorbidities. In such a situation, physicians tend to undervalue the utility and effectiveness of a PDA tool for FH:

“*The people that have this [FH] always have multi-morbidity, so I’m seeing them for you know hyperlipidemia might be number 6 on the problem list that day (chuckles)*” (Physician FG II).

Other physician participants, as well as genetic counselors, argued that in addition to multiple morbidities, patient’s age, as well as health literacy, are important factors that need to be considered for the developing of the tool. One physician emphasizing on the patient’s age factor, stated:

“*I mean, again, they may still live until they’re 80. They may never suffer a heart attack or stroke, and maybe it’s through diet and exercise, maybe it’s, again, pure luck; we won’t know. I’m not saying that you put in there luck is a reason, but we don’t know that part of medicine where …… it needs to be presented in a way if you take a 50-year-old gentleman, someone who has that condition, here’s potentially what literature evidence would show us it looks like; and then for other 50-year-olds, here’s how it would look based on that age group and kind of balancing for people how they can compare and leverage it that way versus—you’re 50 and you have this condition, and this is the only pathway that you can go down is treatment and stuff because otherwise you’re going to end up with a heart attack and stroke. And we know what some of the evidence will show us, but we also know that it’s not an absolute*” (Physician FG I).

### 3.2. Discussion about the Content of PDA Related to FH

The focus group participants also discussed what they would like to see in a PDA for FH. They shared their perspectives on treatment modalities available for FH and inherent FH characteristics that should be considered when developing a decision aid for patients deciding on therapy. We broadly categorized their opinions and recommendations into three major categories: treatment options, cost and insurance, and genetic testing.

#### 3.2.1. Treatment Options

Physicians noted that many FH patients are not aware of the significant risk of cardiovascular disease associated with this condition, and thus they might underestimate the importance of therapy. Therefore, physicians thought that prior to discussing treatment options, it would be ideal to make sure that patients truly understand what FH means and how it differs from other types of hypercholesterolemia. One physician explained:

“*I think that’s the first hard part, as you said, is convincing them this is not just hyperlipidemia, this is different, and getting them to understand that, I think, is the first hard part. And beyond that, you know, I think that’s when, you know, patient preference and all that other stuff comes into play; and as long as they understand basically at a fundamental level what we understand about the disease, then I think they can make an informed decision about it; and whether it’s what we decide or not, that’s their choice*” (Physician FG I).

Physicians also provided insights into their approach to decision-making related to FH treatment. Many physicians emphasized that it is widely accepted that most FH patients should be treated initially with statins. Thus, FH treatment was viewed as less preference-sensitive decision as compared to conditions such as major depressive disorder where multiple medications can be considered as first-line therapy. One physician stated:

“*For a condition (major depressive disorder) where there are half a dozen different equally reasonable first choices, which is not the case here. So I mean 99% of the time if somebody said yes, I think I’d like to take a medication for this, we’d prescribe a statin*” (Physician FG I).

Resonating physicians’ opinions some patients in our focus groups asked us about how FH is characterized and whether the definition of FH has changed over the period. All these basic questions clearly imply the poor level of knowledge and understanding of patients about FH. One patient expressed his doubt about the categorization of FH and differential practice of physicians. He inquired:

“*I’m wondering if the definition of FH has changed over the years. The reason I ask that is because I have had a diagnosis of that, maybe under my previous doctor, which would go back a ways, you know, before he retired; but I actually asked … or it came up fairly recently with my current doctor, and he said I didn’t have a diagnosis of it. Well, maybe that was in the paper record; I don’t know or maybe he just, you know, forgot … nobody’s perfect … so but that’s what made me wonder if there were different criteria*” (Patient FG I).

#### 3.2.2. Cost and Insurance

Most participants in the focus groups acknowledged financial costs and insurance coverage as powerful factors that significantly influence decisions related to diagnosis and treatment modalities. One patient stated:

“*I think unfortunately a lot of times whether or not your insurance is going to cover the cost and costs often overrides the health benefit*” (Patient FG II)

Therefore, for a decision aid to be most helpful, discussants highlighted the importance of presenting approximate costs of treatment options in the tool. One physician described how most patients would like to be provided with such information, saying:

“*But, but if it’s going to affect me and I have a copay or whatever my insurance allows, (and)it doesn’t tell me how much I have to pay as an individual and I want to know that*” (Physician FG II).

Notably, another physician alluded to the pivotal role insurance companies play when patients are considering medical care decisions, noting that it would be equally important for decision aids to encompass information about costs in conjunction with insurance coverage for any presented option.

“*And I would include that insurance may or may not cover for the test because that’s going to be an issue […] and now it spurs my discussion with the patients because they can say, “Well, it’s expensive but my insurance will pick up the tab*” (Physician FG II).

The same physician also hinted to the fact that patients’ perceptions of costs may be altered if they are made aware that insurers will cover for a given test or medication.

Interestingly, genetic counselors also reflected on how patients would perceive costs presented for treatment options. One genetic counselor advised paying particular attention when prices of different options are to be included in a decision aid, as many patients tend to expect the actual costs to be closer to the lower end of any given price range.

“*I would caution because patients get hung up on this. So, if they are given a price range ($500 to $2000), they’ll expect it to be $500*” (Genetic Counselor FG).

#### 3.2.3. Genetic Testing

Given the genetic basis of FH, the different aspects of genetic testing were among the dominant themes that emerged in the discussions. Many patients were not even aware of the existence of a genetic test for FH with one patient responded to a question about genetic testing: *“it’s a mystery!“.* However, most patient participants expressed their willingness to learn more about the genetic nature of FH and how genetic testing is different from other diagnostic tests. One patient stated:

“*And to the point where maybe explaining what genetic testing is and how that is relevant to the conversation to a layperson would be helpful. Just a little bit more about what genetic testing is and how it’s different from doing that clinical diagnosis based on just lab test values*” (Patient FG I).

Physicians discussed various aspects of genetic testing including the complexity of interpreting results, indications for testing, and financial considerations. One physician, as many others, expressed the unease with which physicians deal with genetic test results, largely owing to relative lack of knowledge and inadequate training in the field of genetics:

“*This is more complex than offering a test and not offering a test, because the testing is easy I mean you can go for a blood test and get, but then the interpretation part is where the complexity comes in. If it comes back to us now you interpret this; I don’t think I’m adequately trained or know how to relate this back to the patient in terms of that particular test result as it applies to that patient, to the patient’s family*” (Physician FG I).

Another challenging aspect of genetic testing for physicians was explaining the relevance of such a test for FH patients, since they felt that results may not significantly alter individual management plans. One physician elaborated on this notion by stating:

“*I’m probably bringing it up in the context of what it will do for family members rather than the individual patient. Um, because I’m going to treat the individual patient about the same whether they you know actually have the gene or not […] But, that has an implication for should we be screening family members at an earlier age? Should we be doing different things with family members?*” (Physician FG II).

Many physicians viewed that the decision on whether to undergo genetic testing or not is heavily influenced by insurance coverage and incurred costs. One physician explained that patients with limited resources would likely prioritize spending on essential medications over investing in expensive supplementary testing. He further says:

“*I think it’s also important to put that cost in perspective, I mean that cost can buy an awful lot of statins. And um some of our patients are having problems paying for the statins and so if that’s the case; it doesn’t make sense to do the genetic testing in somebody who’s having trouble affording the statin. You know cause this is years’ worth of statins that we’re burning up with this genetic test in terms of cost*” (Physician FG II).

Genetic counselors, on the other hand, were more familiar with the “ins” and “outs” of genetic testing for FH. However, they also acknowledged that the outcomes of genetic testing might be less straightforward to interpret compared with other diagnostic and screening modalities. One genetic counselor stated:

“*I have a lot of experience with FH both in seeing patients and then also you know going through the literature and reading about this condition. But one thing I know that it was more you know the question of when I started out was you know why is genetic testing helpful for these people? What are the motivations of why people do this genetic testing besides just to confirm the diagnosis? And that is something that I’ve gotten more of through reading the literature and through um you know talking with other counselors who counsel for this particular condition*” (Genetic Counselor FG).

Given the uncertainties surrounding many aspects of genetic testing, most genetic counselors were in favor of developing decision aids that inform patients making a decision on genetic testing. One genetic counselor clarified:

“*I think that would be one nice piece if you know talking through what are the potential benefits of genetic testing, the risks of that and having that lined out in some sort of tool almost so the patient can kind of compare both risks and benefits*” (Genetic Counselor FG).

### 3.3. Shared Decision-Making

The potential role of decision aids in creating an environment for SDM was discussed in all focus groups. Physicians noted that when used properly, decision aids might provide additional time for discussion during consultations for SDM to occur. One physician also emphasized that while PDAs ideally help patients make decisions more concordant with their goals and values, caution should be exercised not to use such tools persuasively to impose clinicians’ views that do not necessarily match patients’ preferences. This physician stated:

“*It creates the picture that we’re involving you, we’re giving you all this information, we’re engaging you in a discussion, but we’re really not. We’re trying to convince them that what we think is right is best for them, and you should do what we want*” (Physician FG I).

Most patients voiced their eagerness to participate in the decision-making process alongside their physicians. One patient said:

“*I really prefer to be involved in it and have my opinion asked and maybe it’s not valid but I love it when they can pull up on a screen and go ‘Well, these are what the results have been.’*” (Patient FG II).

Another patient echoed this idea, noting that before taking any medication, patients would like physicians to provide more information related to drug side effects and costs:

“*My doctor was telling me… we’re going to put you on some kind of statin; I would want to read all about the statin and … I’d just want to know what the side effects were for the statin, what you know, the cost, everything*” (Patient FG I).

Interestingly, one physician noted that not all patients are willing to actively participate in making health care decisions and they might prefer to entrust this task to their treating physicians. The physician described this conversation when the patient says:

“*Well (doctor), if you were in my shoes what would you do?” then the physician would say “Well, you know here’s what I would recommend*” (Physician FG II).

## 4. Discussion

FH is an inherited disorder associated with increased cardiovascular risk that requires patients to make multiple, potentially impactful decisions regarding the management of their condition. Possible factors that can contribute to the complexity of the decision-making process for FH patients include the genetic nature of the disease, evidence gaps in different aspects of care, the need for lifelong medications, and numerous medically reasonable options for screening and treatment [8]. By fostering SDM, PDAs can help patients make decisions that are aligned with their goals and values. As an initial step towards developing PDAs for FH, we aimed to first explore the perspectives of relevant stakeholders, namely patients, physicians, and genetic counselors, about PDAs pertaining to FH; and second, to identify themes that patients and clinicians want to incorporate in a PDA related to FH.

Participants provided valuable input on how to guide the development process of a PDA for FH. Across all focus groups with clinicians and patients, emphasis was placed on the language format that will be used in a PDA. In concordance with the International Patient Decision Aids Standards (IPDAS) [14], using plain language was viewed as an essential requirement for a successful PDA implementation. In addition, while a few participants seemed to be agnostic to the logistics of tool delivery during encounters, most discussants stressed the importance of taking into account factors that can maximize the accessibility of these tools. Frequently, PDAs are tools that are used during clinical encounters with both the clinician and the patient involved simultaneously [15]. However, given the time constraints inherent to clinical encounters with the average encounter lasting only 15 min [20], and the abundance of information patients may need to make an informed decision, a format that enables patients to access those tools both before and after their consultation sessions with healthcare providers was advocated by our participants as a potential solution.

Barriers to implementation varied across different participant groups. While physicians were enthusiastic about providing their patients with the best information available, they were concerned about time constraints. These findings resonate with previous studies that examined the feasibility of SDM implementation in the healthcare setting [21,22,23]. This implies that for a PDA to be adopted by physicians, developers should take these concerns into consideration early in the development process. Additionally, several studies suggest that physicians might be more motivated about integrating SDM strategies into their clinical workflow if health care policymakers provide the financial incentive for such modifications to occur [24]. Direct reimbursement for time spent using PDAs for SDM might help relieve the tension between physicians’ willingness to involve patients in the decision-making process and constraints on their time. Inexperience with FH was another major barrier. A recent qualitative study demonstrated that even experienced cardiologists were not comfortable making management plans for FH patients and they would rather refer those patients to specialty clinics [25].

Other studies highlighted that clinicians’ uncertainty about the evidence presented in decision aids might affect the use of such tools [26]. Remarkably, such a concern was not raised by participants during discussions. This could be due to the relative unfamiliarity with FH as a condition, which might render clinicians less able to critically evaluate evidence related to FH, and consequently less focused on the quality of evidence.

In contrast, compared to physicians, genetic counselors were not particularly concerned about time given the relatively time-lenient clinical encounters. Although patients who participated in our focus group discussions tended to be well-educated with 11 out of 13 patients having attended college or graduate school, inadequate health literacy about FH seemed to be the main barrier for patients. From our discussions, it became apparent that patients frequently dismiss FH as “just high cholesterol” with many patients being unaware of the excessive ASCVD risk and familial implications associated with their condition. According to clinicians, this commonly held misconception is particularly alarming when discussing treatment options with FH patients since many patients might consequently underestimate the importance of lipid-lowering therapy.

This gap in knowledge about FH among patients has been previously reported with one study finding that 57% of patients were not at all familiar with FH despite being aware of having high cholesterol [6]. However, given that PDAs can have both educational and decisional support aspects, this finding can also be viewed as an opportunity for PDAs rather than a challenge. It has been well documented that when used properly, PDAs are better than usual care in improving patients’ knowledge and can empower patients to make more-informed decisions [15,26].

Genetic testing was discussed among participants in great detail. The decision of whether to proceed with genetic testing requires prudent evaluation of multiple factors. Patients appeared to be largely uninformed regarding options for genetic testing. Nevertheless, patient participants expressed great interest in learning more about the genetic components of their condition.

Apparent physician uncertainty surrounding genetic information and interpreting genetic test results suggests a need for tools capable of facilitating mutual understanding of genetic testing for both physicians and their patients. Genetic testing for conditions such as FH is becoming accessible and affordable to patients, and as such, it is anticipated that physicians will encounter questions from patients regarding genetic testing. The primary value of genetic testing may lie in facilitating early detection and treatment of first-degree relatives of patients, particularly children. On the other hand, concerns associated with genetic testing might include insurance coverage, stigmatization, and alteration in individual management plans. Given the numerous considerations associated with genetic testing, PDAs can provide a platform for thoughtful evaluation of the risks and benefits of genetic testing within the context of patient’s values and preferences. As such, PDAs can better inform patients and providers and help individualize the decision to undergo the genetic testing process.

A limitation of our study is that the focus group discussions with physicians included only two cardiologists. Acknowledging that cardiologists tend to have more experience in managing patients with FH compared to internists and family physicians, the perception of clinicians’ inexperience with FH suggested by our study might have been overestimated. Another potential limitation is that most of our patient participants were relatively well-educated and our findings may not reflect the perceptions of patients with lower education levels. Compared to patient participants in our study, patients with FH belong to a more diverse population with respect to age, race, ethnicity, and level of education. Further studies that elicit perspectives of a more diverse group of patients could provide valuable insight into the development process for PDAs.

## 5. Conclusions

In conclusion, our study elicited valuable insights from various stakeholders—physicians, genetic counselors, and patients—regarding PDAs for FH. In general, stakeholders were in favor of developing tools which can inform and individualize discussion about genetic testing and treatment options for FH. Physicians valued a tool that facilitates knowledge transfer to FH patients who may or may not be aware of the excessive cardiovascular risk associated with their condition. Patients desired a tool to help them understand the genetic aspects of and treatment options related to FH. Genetic counselors emphasized the inclusion of visual aids to support discussion with patients. We also identified potential barriers to and facilitators of PDA implementation. Facilitators include adapting plain language, emphasizing an accessible tool format, and integrating visual aids into the tool. Barriers to implementation were mainly related to physicians’ time constraints and relative inexperience with FH. The input of various stakeholders will inform the development of a prototype tool that will be iteratively tested before implementation in the clinical setting.

## Figures and Tables

**Table 1:** Sample questions from focus group discussions with clinicians.

*Counseling patients with FH* Suppose you have a patient with suspected FH: In your opinion, what would have made explaining the genetic aspects of the disease easier? Would a patient decision aid (a document designed to be used with your patient that attempts to take patient preferences into account when deciding on genetic testing for FH mutations) have helped you?
*Creating a Patient Decision Aid* What is your opinion of shared decision-making? Shared decision-making with the use of a patient decision aid? What has been your experience with using patient decision aids? What do you like about them? What do you dislike about them? How have they been most helpful for you? For the patient? What was your experience like when you were counseling patients for genetic testing of the disease? Did you feel you gave a sufficient explanation of the available options? Were you comfortable with giving advice to the patient regarding the various options?

**Table 2:** Sample questions from focus group discussions with patients.

*FH Diagnosis* When you first heard you were diagnosed with FH: What was your reaction to the initial diagnosis? What was the effect on your spouse, your children, or significant others? How was the diagnosis presented to you initially? Was the explanation you received about the diagnosis adequate? What was lacking? What do you wish would have been done differently? What did you like about the way FH was explained to you? In your opinion, what would have made understanding this disorder or disease easier? How would a patient decision aid (a document) designed to be used with your doctor that attempts to take your preferences into account when choosing an initial therapy) have helped/not helped you (in your opinion)? Would a visual aid demonstrating the effects of various therapies have helped/not helped you?
*Creating a Patient Decision Aid* What do you think of when you hear the term patient decision aid (PDA)? What was your experience like when you were given various options for management of the disease? How did your doctor explain the different treatment options available to you? Or did your doctor just discuss a single medication? What was your impression of the style of decision-making that occurred? Would you have preferred that your doctor explained all the different treatment options available to you? Were your values incorporated into the decision-making process? By values, we mean what you were most interested in gaining from treatment and what you were most fearful of in initiating treatment Could you share an example of this with us (of either being asked for your values or not being asked for your values)?

**Table 3:** Demographic characteristics of clinicians interviewed (*n* = 17).

Clinicians	*n* (%)
Sex (females)	11 (64.7)
Age *	
<40	10 (66.6)
40–60	4 (26.6)
>60	1 (0.06)
Race (White)	17 (100)
Years of experience	
<5	8 (47.0)
5–10	5 (29.4)
>10	4 (23.5)

* Data missing from 2 (*n* = 15).

**Table 4:** Demographic characteristics of patients interviewed (*n* = 14).

Patients	*n* (%)
Sex (females)	9 (64.2)
Age	
<40	0 (0)
40–60	7 (50)
>60	7 (50)
Race (White)	14 (100)
Marital status (married)	8 (57.1)
Education *	
High school or less	2 (15.3)
Any college	7 (53.8)
Graduate school	4 (30.7)

* Data missing from 1 (*n* = 13).
